# Antenatal bleeding: Case definition and guidelines for data collection, analysis, and presentation of immunization safety data

**DOI:** 10.1016/j.vaccine.2017.01.081

**Published:** 2017-12-04

**Authors:** Malavika Prabhu, Linda O. Eckert, Michael Belfort, Isaac Babarinsa, Cande V. Ananth, Robert M. Silver, Elizabeth Stringer, Lee Meller, Jay King, Richard Hayman, Sonali Kochhar, Laura Riley, for The Brighton Collaboration Antenatal Bleeding Working Group

**Affiliations:** a Department of Obstetrics and Gynecology, Massachusetts General Hospital, Boston, MA, USA; b Department of Obstetrics and Gynecology, University of Washington, Seattle, WA, USA; c Department of Obstetrics and Gynecology, Baylor College of Medicine, Houston, TX, USA; d Department of Obstetrics and Gynecology, Texas Children’s Hospital, Houston, TX, USA; e Sidra Medical and Research Center/Weill Cornell Medicine-Qatar/Women’s Hospital, Qatar; f Department of Obstetrics and Gynecology, College of Physicians and Physicians, Columbia University, New York, NY, USA; g Department of Epidemiology, Joseph L. Mailman School of Public Health, Columbia University, New York, NY, USA; h Department of Obstetrics and Gynecology, University of Utah, Salt Lake City, UT, USA; i Department of Obstetrics and Gynecology, University of North Carolina at Chapel Hill, NC, USA; j Gloucestershire Hospitals NHS Foundation Trust, UK; k SanofiPasteur, Swiftwater, PA, USA; l Department of Obstetrics and Gynaecology, Gloucestershire Hospital, Gloucester, UK; m Global Healthcare Consulting, India; n Erasmus University Medical Center, Rotterdam, The Netherlands

**Keywords:** Antenatal bleeding, Adverse event, Immunization, Guidelines, Case definition

## 1 Preamble

### 1.1 Need for developing case definitions, and guidelines for data collection, analysis, and presentation for antenatal bleeding as an adverse event

Bleeding in the second and third trimesters of pregnancy affects 6% of all pregnancies, and has distinct etiologies from first-trimester bleeding [1]. In the vast majority of cases, antenatal bleeding is vaginal and obvious; however, rarely, it may be contained within the uterine cavity, the intraperitoneal space, or the retroperitoneal space. The etiologies of antenatal bleeding, also referred to as antepartum hemorrhage, are heterogeneous. In cases of severe antepartum hemorrhage, complications include preterm delivery, cesarean delivery, blood transfusion, coagulopathy, hemodynamic instability, multi-organ failure, salpingectomy/oophorectomy, peripartum hysterectomy, and in some cases, either perinatal or maternal death.

The goal of this Working Group was two-fold:

(1) to define sources of pathologic antenatal bleeding in the second or third trimester of pregnancy that are directly attributable to pregnancy and are either common and/or catastrophic;
(2) to define each source of antenatal bleeding for the purposes of future case ascertainment.

The charge to the Brighton Collaboration Working Groups to define various adverse obstetric and pediatric events includes an aim to more easily identify immunization-related adverse events. In the case of antenatal bleeding, our Working Group felt strongly that there is no biologic plausibility or mechanistic explanation linking immunizations to antenatal bleeding. Moreover, as immunizations and antenatal bleeding are common occurrences in the course of any individual pregnancy, it is quite likely that these events will co-occur without suggesting causation. To date, there is one case report of antenatal bleeding occurring in a pregnancy where a tetanus, diphtheria, and acellular pertussis vaccination was also administered [2]. However, the definition used to identify the antenatal bleeding event is not clearly presented. Standardized definitions across trials, surveillance systems, or clinical settings will facilitate case ascertainment and analysis of potential risk factors for antenatal bleeding.

In this document, we focus on placenta previa, morbidly adherent placentation, vasa previa, placental abruption, cesarean scar pregnancy, intra-abdominal pregnancy, and uterine rupture as important sources of antenatal bleeding. Cesarean scar pregnancy and intra-abdominal pregnancy are rarely listed as causes of antenatal bleeding in the second and third trimester. Nonetheless, we included these causes as they are more likely to result in late presentation with a high risk of heavy maternal bleeding in settings in which ultrasound diagnosis of pregnancy is limited or unavailable.

Another common source of bleeding is labor, whether at term or preterm. Although preterm labor is pathologic and addressed in another document [3], bleeding in the context of labor alone is not. This is not addressed in our document. Non-obstetric genital tract bleeding may also occur during pregnancy, including neoplastic, infectious, traumatic, or iatrogenic causes. Urinary tract infections or hemorrhoids may also be misidentified as antenatal bleeding until additional workup is performed. This document will focus solely on the pregnancy-attributable etiologies of antenatal bleeding.

### 1.2 Methods for the development of the case definition, and guidelines for data collection, analysis, and presentation for antenatal bleeding as an adverse event

Following the process described in the overview paper [4] as well as on the Brighton Collaboration Website http://www.brightoncollaboration.org/internet/en/index/process.html, the Brighton Collaboration *Antenatal Bleeding Working Group* was formed in 2016 and includes members with a diverse background in clinical experience, location of practice, and scientific expertise in sources of antenatal bleeding. The composition of the working and reference group as well as results of the web-based survey completed by the reference group with subsequent discussions in the working group can be viewed at: http://www.brightoncollaboration.org/internet/en/index/working_groups.html.

To guide decision-making for case definitions, a literature search was performed in PubMed, including the following terms: pregnancy, antenatal bleeding, antepartum bleeding, antepartum hemorrhage, placenta previa, vasa previa, abruptio placenta, placenta accreta, morbidly adherent placenta, abdominal pregnancy, cesarean scar pregnancy, uterine rupture, abdominal pregnancy, intra-abdominal pregnancy, and vaccination. Major obstetric textbooks and published guidelines from major obstetric societies throughout the world were also surveyed. This review resulted in a detailed summary of 33 articles used to establish case definitions for antenatal bleeding. The search also resulted in the identification of 1 reference containing information regarding vaccination administration and antenatal bleeding (as defined by the listed PubMed search terms above).

### 1.3 Description of sources of antenatal bleeding

We first begin with a brief description of each etiology, the underlying pathophysiology, incidence, and risk factors. For most conditions, incidence data are derived from settings in which the condition has been most systematically studied, often North America and Western Europe. Incidence data not derived from these areas is specified in the following paragraphs.

#### 1.3.1 Placenta previa

Placenta previa occurs when the placenta partially or completely overlies the internal cervical os. This is in contrast with low-lying placenta, in which the placenta lies within 2 cm of the internal cervical os but does not extend across it. The etiology of placenta previa is unknown. Risk factors include smoking, advanced maternal age, multiparity, in vitro fertilization, multiple gestation, Asian race, prior endometrial damage, prior pregnancy termination or spontaneous abortion, prior cesarean delivery, and prior placenta previa [1], [5], [6]. These risk factors suggest that the pathogenesis may be driven by endometrial damage or suboptimal endometrial perfusion in other areas of the uterus. The incidence of placenta previa at term is approximately 1 in 200 pregnancies; the incidence is higher earlier in gestation, but many placenta previas resolve as the lower uterine segment develops and the placenta preferentially expands towards more vascularized areas of the uterus [1], [5].

#### 1.3.2 Morbidly adherent placentation

Morbidly adherent placentation occurs when the placenta implants abnormally into the uterine myometrium, rather than the normal implantation of the placenta into the uterine decidua basalis [1], [5], [7]. Invasive placentation occurs as a result of the absence of the decidua basalis and incomplete development of or injury to Nitabuch’s layer [1], [5], [8]. The incidence of morbidly adherent placentation is 1 in 300 to 1 in 500 pregnancies [5]. The most significant risk factor is placenta previa in the context of one or more prior cesarean deliveries, or other uterine surgery. With one prior cesarean delivery and a placenta previa, the risk is 11%; with 3 or more cesarean deliveries and a placenta previa, the risk is greater than 60% [9]. Other common risk factors include advanced maternal age, advanced parity, cesarean scar pregnancy, and in vitro fertilization [5], [7], [10], [11], [12].

#### 1.3.3 Placental abruption

Placental abruption occurs when the placenta detaches prematurely from its implantation site. Traditionally conceptualized as primarily an “acute” event often resulting from physical trauma to the abdomen, contemporary data suggest that placental abruption is often chronic [13], [14], [15], [16], [17]. Nevertheless, acute placental abruptions still occur. Abruptions may either be revealed, with vaginal bleeding as an early symptom, or concealed, with blood remaining trapped within the uterus. Pathophysiologic mechanisms involved in abruption include uteroplacental underperfusion, ischemia, placental infarctions, and chronic hypoxia [18], [19], [20]. In very rare circumstances abruption can follow second trimester diagnostic and therapeutic intrauterine procedures (amniocentesis, CVS, fetal surgery). Abruption affects about 1% of pregnancies, but is associated with a recurrence risk of about 10–15% for one prior abruption, 20–30% after two, and ≥30% after three or more abruptions [21], [22]. Other risk factors include first trimester bleeding, hypertension, thrombophilia, illicit drug use (especially cocaine), smoking, trauma, in vitro fertilization, and premature rupture of membranes [23], [24], [25], [26]. Pregnancies diagnosed with abruption end 3–4 weeks earlier than other pregnancies, with well over half delivering preterm. This is in contrast to a preterm birth rate of 12% among unaffected pregnancies [26], [27], [28], [29].

#### 1.3.4 Vasa previa

Vasa previa occurs when fetal blood vessels course within the amniotic membranes across the internal cervical os or within 2 cm of the os. Type I vasa previa occurs with a velamentous umbilical cord insertion into the membranes, consequently allowing for fetal vessels to run free within the membranes between the umbilical cord and placenta. Type II vasa previa occurs with the development of a succenturiate placental lobe and main placental lobe, connected by fetal vessels that freely course within the membranes. Vasa previa is rare, with an incidence of 1 in 2500 deliveries. Risk factors include resolved low-lying placenta, placenta previa, and multiple gestation [5], [30].

#### 1.3.5 Cesarean scar pregnancy

A cesarean scar pregnancy is an ectopic pregnancy implanted in a previous cesarean (hysterotomy) scar, surrounded by myometrium and connective tissue. This occurs due to a small defect in the cesarean scar, as a result of poor healing and poor vascularization of the lower uterine segment with resultant fibrosis [31]. The pathophysiology of cesarean scar pregnancies is similar to an intrauterine pregnancy with morbidly adherent placentation [32]. Cesarean scar pregnancies occur in about 1 in 2000 pregnancies and account for 6% of ectopic pregnancies among women with a prior cesarean delivery [31]. As the recognition of cesarean scar pregnancies is relatively recent, risk factors are not yet clear; however, as with morbidly adherent placentation, the incidence appears to correlate with the number of prior cesarean deliveries [32].

#### 1.3.6 Intra-abdominal pregnancy

Intra-abdominal pregnancy is a rare form of an ectopic pregnancy, in which a pregnancy implants into the peritoneal cavity or abdominal organs. Most commonly, this occurs due to tubal ectopic pregnancy with tubal extrusion or rupture and secondary implantation; primary implantation into the peritoneal cavity is also possible. Pregnancies may be asymptomatic, or may present with life-threatening intra-abdominal hemorrhage. The incidence is difficult to ascertain, as data are derived from case reports, but is reported to be 1–2 in 10,000. Risk factors are artificial insemination, in vitro fertilization, uterine surgeries, and prior tubal or cornual pregnancy [33], [34].

#### 1.3.7 Uterine rupture

Uterine rupture is the complete nonsurgical disruption of all layers of the uterus. Uterine rupture may occur either in an unscarred uterus or at the site of a prior hysterotomy scar. The incidence of rupture of the unscarred uterus is approximately 1 in 20,000 deliveries in high-resource settings, but can be as high as 1 in 100 deliveries in low-resource settings, where the majority of this type of rupture occurs [35], [36], [37]. Risk factors for uterine rupture in an unscarred uterus include a contracted pelvis, prolonged dystotic labor, multiparity, morbidly adherent placentation, malpresentation, use of strong uterotonic drugs perhaps with cephalopelvic disproportion, operative vaginal deliveries at high station, and congenital weakness of the myometrium [35]. In high-resource settings, uterine rupture most commonly occurs in the context of a prior hysterotomy scar or transfundal surgery [37]. The incidence of this event ranges from approximately 1 in 200 up to 1 in 10, depending on the type of hysterotomy and the use of labor augmentation [38], [39]. Additional risk factors include the number of prior cesarean deliveries, interdelivery interval less than 18 months, one-layer uterine closure, and open fetal surgery [40], [41], [42].

### 1.4 Rationale for selected decisions about case definitions for antenatal bleeding as an adverse event

#### 1.4.1 Formulating case definitions that reflect diagnostic certainty: weighing specificity versus sensitivity

The number of signs, symptoms, and diagnostic tests that will be documented for each case may vary considerably. The case definition has been formulated such that the Level 1 definition is highly specific for the condition. As maximum specificity normally implies a loss of sensitivity, an additional diagnostic level has been included in the definition to increase sensitivity while retaining an acceptable level of specificity. In this way, it is hoped that all possible cases of antenatal bleeding can be systematically captured.

The grading of definition levels is about diagnostic certainty, not clinical severity of an event. Thus, a clinically very severe event may appropriately be classified as Level 2 or 3 rather than Level 1 if it could reasonably be of an alternative etiology – either another cause of antenatal bleeding, or unrelated to antenatal bleeding entirely. Detailed information about the severity of the event should always be recorded, as specified by the data collection guidelines [43].

#### 1.4.2 Rationale for individual criteria or decision made related to the case definitions

##### 1.4.2.1 Pathology findings

In certain cases, pathologic findings serve as the gold standard to confirm the presence of a pathologic entity. This is the case for morbidly adherent placentation, where the surgical specimen is often the hysterectomy specimen with placenta in-situ. Pathologic findings for cesarean scar pregnancy managed by hysterectomy with the gestational sac in-situ is also the gold standard for diagnosis; however, a hysterectomy is not always performed, and histologic confirmation may not be possible. Histological findings identify many but not all cases of placental abruption. The other etiologies of antenatal bleeding included within this document do not lend themselves to a histologic diagnosis.

##### 1.4.2.2 Laboratory findings

No specific laboratory findings were included in case definitions of antenatal bleeding, as none of these clinical entities are associated with specific or identifiable laboratory parameters. Anemia and coagulopathy associated with significant antenatal bleeding are to be diagnosed and managed using usual clinical algorithms.

##### 1.4.2.3 Radiology findings

Ultrasound findings in a pregnancy complicated by antenatal bleeding are highly important in identifying and differentiating several conditions and thus are included in many case definitions. MRI findings may be used in some circumstances when this modality is available. See below regarding safety data.

#### 1.4.3 Safety of imaging in pregnancy

Prenatal ultrasound uses sound waves passing through an acoustic window to visualize deeper tissue and structures, including a fetus. Ultrasound is considered safe in pregnancy, and there have been no reports of adverse fetal or neonatal outcomes from prenatal ultrasound imaging. Applying the ALARA (As Low As Reasonably Achievable) principle is recommended during diagnostic imaging procedures [44], [45]. Magnetic resonance imaging (MRI) technology has also been used in pregnancy for several indications after inconclusive or nondiagnostic prenatal ultrasound. There has never been any documented fetal or neonatal harm and the procedure is considered safe in pregnancy. While the quality of imaging may be superior with gadolinium-enhanced imaging, its use is not currently recommended in pregnancy due to theoretical harms. Nonetheless, clear harm from gadolinium has not been demonstrated [45].

#### 1.4.4 Timing of adverse event with relation to timing of immunization

As noted in the preamble of this document, both immunizations and antenatal bleeding are common events in pregnancy. We feel strongly there is no current evidence or biological plausibility to suggest a causal link between immunization and antenatal bleeding. In order to appropriately assess this question, pregnant women who are and are not exposed to immunizations would need to be prospectively studied to identify any association with antenatal bleeding. However, withholding immunizations in pregnancy would not be ethical, and thus we are left with case reports and other epidemiologic studies of association that may lead to inappropriate conclusions.

#### 1.4.5 Differentiation from other associated disorders

As previously discussed, the focus of this Working Group is to define pathologic primary causes of antenatal bleeding. Labor, whether at term or preterm, may present with vaginal bleeding, yet in this instance, the pathway of preterm labor is the primary pathologic event. The Brighton Working Group on Pathways of Preterm Birth describes the pathophysiology of preterm labor in detail [3].

### 1.5 Guidelines for data collection, analysis and presentation

As mentioned in the overview paper, the case definition is accompanied by guidelines that are structured according to the steps of conducting a clinical trial, i.e. data collection, analysis and presentation. Case definitions and guidelines are not intended to guide or establish criteria for management of ill infants, children, or adults. Both were developed to improve data comparability.

### 1.6 Periodic review

Similar to all Brighton Collaboration case definitions and guidelines, review of the definition with its guidelines is planned on a regular basis (i.e. every three to five years) or more often if needed.

## 2 Case definition of antenatal bleeding^3^

### 2.1 For all levels of diagnostic certainty

Antenatal bleeding is a clinical syndrome characterized by bleeding in the second or third trimester of pregnancy. Pathologic etiologies attributable to the pregnant state include placenta previa, morbidly adherent placenta, vasa previa, placental abruption, cesarean scar pregnancy, intra-abdominal pregnancy, and uterine rupture.

For both levels of diagnostic certainty for each etiology of antenatal bleeding:

• The patient is determined to be in the second or third trimester of pregnancy (refer to Brighton Working Group document to establish dating in pregnancy [46]).
• Bleeding is either documented vaginally or suspected to be occurring intrauterine, intraperitoneally, or (rarely) retroperitoneally, based on clinical signs and symptoms.
• In the case of ultrasound-based diagnosis, transvaginal ultrasound is more specific than transabdominal ultrasound, and transvaginal ultrasound is recommended where available.

For each definition, the diagnostic levels reflect diagnostic certainty and must not be misunderstood as reflecting different grades of clinical severity. Moreover, defining levels of clinical severity of antenatal bleeding is beyond the scope of this document.

#### 2.1.1 Placenta previa

**Table:** 

**Level 1** – Second or third trimester ultrasound (and/or MRI) evidence of placental tissue overlying or abutting the internal cervical os.

**Level 2** – Painless vaginal bleeding in the second or third trimester,
AND a high presenting part or abnormal fetal lie,
AND one of the following:
EITHER a pelvic exam with fullness palpable in the fornices (avoiding digital cervical exam) OR a speculum exam with tissue visible through an open cervical os

#### 2.1.2 Morbidly adherent placentation

**Table:** 

**Level 1** – There are two definitions of equal specificity.
Second- or third-trimester ultrasound or MRI evidence of placenta previa,
AND one of the following ultrasound features noted in Table 1,
AND one of the risk factors as noted in Table 2.

OR

Morbidly adherent placentation found on histology in a hysterectomy or partial wedge resection specimen.

**Level 2** – There are two definitions of equal specificity.
Ultrasound evidence of placenta previa,
AND hypervascularity at the site of the uteroplacental interface, diagnosed at laparotomy.

OR

Difficulty with placental separation after delivery of the infant, at either a vaginal or cesarean delivery with resultant hemorrhage due to partial separation.

**Table 1:** Ultrasound features of morbidly adherent placentation [47].

Greyscale	Loss of the retroplacental sonolucent zone
	Irregular retroplacental sonolucent zone
	Thinning or disruption of the hyperechoic serosa–bladder interface
	Presence of focal exophytic masses invading the urinary bladder
	Abnormal placental lacunae

Color doppler	Diffuse or focal lacunar flow
	Vascular lakes with turbulent flow (peak systolic velocity over 15 cm/s)
	Hypervascularity of serosa–bladder interface
	Markedly dilated vessels over peripheral subplacental zone

3D Power doppler	Numerous coherent vessels involving the whole uterine serosa–bladder junction (basal view)
	Hypervascularity (lateral view)
	Inseparable cotyledonal and intervillous circulations, chaotic branching, detour vessels (lateral view)

**Table 2:** Risk factors for morbidly adherent placentation.

Prior cesarean delivery
Prior uterine surgery (including endometrial ablation or dilation and curettage)
Cesarean scar pregnancy

#### 2.1.3 Vasa previa

**Table:** 

**Level 1** – Second trimester ultrasound evidence of fetal vessels (vessel with fetal heart rate identified by color flow Doppler) running through the membranes and overlying the internal cervical os,
AND post-delivery examination of the placental specimen with unsupported fetal vessels within the membranes.

**Level 2** – Vaginal bleeding in the second or third trimester at the time of ruptured amniotic membranes,
AND fetal heart rate changes ultimately resulting in sinusoidal rhythm/terminal bradycardia,
AND delivery of a pale, anemic infant or recent stillbirth or neonatal death [48],
AND post-delivery examination of the placental specimen with unsupported fetal vessels within the membranes.

#### 2.1.4 Placental abruption

**Table:** 

**Level 1** – There are two definitions of equal specificity.
In the absence of placenta previa on ultrasound, vaginal bleeding in the second or third trimester,
AND one of the following:
EITHER uterine irritability^a^ or labor,
OR clinical signs of hypovolemic shock or coagulopathy.

OR

Placental pathology with histologic findings of a chronic abruption.

**Level 2** – There are two definitions of equal specificity.
Vaginal bleeding in the second or third trimester,
AND uterine irritability or labor, without clinical signs of hypovolemic shock or coagulopathy,

OR

Vaginal bleeding in the second or third trimester,
AND clinical evidence of retroplacental clot or visually evident placental infarcts at the time of delivery.

a Uterine irritability: irregular, frequent uterine activity, not coalesced into clear contractions in a regular pattern.

#### 2.1.5 Cesarean scar pregnancy

**Table:** 

**Level 1** – There are two definitions of equal specificity.
Transvaginal ultrasound with the following characteristics:
• empty uterine cavity, AND • empty cervical canal, without contact with the gestational sac, AND • presence of gestational sac, +/− fetal pole, +/− cardiac activity, in the anterior uterine segment adjacent to the cesarean scar, AND • absence or defect in myometrium between bladder and gestational sac, AND • gestational sac well perfused on Doppler ultrasound (to differentiate from an expulsing, avascular gestational sac).

OR

Hysterectomy specimen with evidence of pregnancy implanted into the cesarean scar.

**There is no Level 2 definition for this condition.**

#### 2.1.6 Intra-abdominal pregnancy

**Table:** 

**Level 1** – At laparotomy, a fetus found within the abdominal cavity, without evidence of uterine rupture, and with placentation not within the uterine cavity.

**There is no Level 2 definition for this condition.**

#### 2.1.7 Uterine rupture

**Table:** 

**Level 1** – Complete uterine disruption at the time of laparotomy in the context of vaginal or intra-abdominal bleeding.

**There is no Level 2 definition for this condition.**

## 3 Guidelines for data collection, analysis and presentation of antenatal bleeding

It was the consensus of the Brighton Collaboration *Antenatal Bleeding Working Group* to recommend the following guidelines to enable meaningful and standardized collection, analysis, and presentation of information about antenatal bleeding. However, implementation of all guidelines might not be possible in all settings. The availability of information may vary depending upon resources, geographical region, and whether the source of information is a prospective clinical trial, post-marketing surveillance or epidemiological study, or an individual report of antenatal bleeding. Also, as explained in more detail in the overview paper in this volume, these are intended as guidelines and are not to be considered a mandatory requirement for data collection, analysis, or presentation.

### 3.1 Data collection

These guidelines represent a desirable standard for the collection of available data following immunization to allow for comparability of data, and are recommended as an addition to data collected for the specific study question and setting. They are not intended to guide the primary reporting of antenatal bleeding for a surveillance system or study monitor. Investigators developing a data collection tool based on these data collection guidelines also need to refer to the criteria in the case definition, which are not repeated in these guidelines.

Guidelines numbers below have been developed to address data elements for the collection of adverse event information as specified in general drug safety guidelines by the International Conference on Harmonization of Technical Requirements for Registration of Pharmaceuticals for Human Use [49], and the form for reporting of drug adverse events by the Council for International Organizations of Medical Sciences [50]. These data elements include an identifiable reporter and patient, one or more prior immunizations, and a detailed description of the adverse event of antenatal bleeding. Additional guidelines have been developed as direction for the collection of additional information to allow for a more comprehensive understanding of antenatal bleeding [4], [43].

#### 3.1.1 Source of information/reporter

For all cases and/or all study participants, as appropriate, the following information should be recorded:

(1) Date of report.
(2) Name and contact information of person reporting^4^ and/or diagnosing the antenatal bleeding as specified by country-specific data protection law.
(3) Name and contact information of the investigator responsible for the subject, as applicable.
(4) Relation to the patient (e.g., immunizer or health care provider [clinician, nurse], family member [indicate relationship], other).

#### 3.1.2 Vaccinee/control

##### 3.1.2.1 Demographics

For all cases and/or all study participants, as appropriate, the following information should be recorded:

(5) Case/study participant identifiers (e.g. first name initial followed by last name initial) or code (or in accordance with country-specific data protection laws).
(6) Date of birth, age, and sex.
(7) For infants: Gestational age and birth weight.

##### 3.1.2.2 Clinical and immunization history

For all cases and/or all study participants, as appropriate, the following information should be recorded:

(8) Past medical history, including hospitalizations, underlying diseases/disorders, pre-immunization signs and symptoms including identification of indicators for, or the absence of, a history of allergy to vaccines, vaccine components or medications; food allergy; allergic rhinitis; eczema; asthma.
(9) Any medication history (other than treatment for the event described) prior to, during, and after immunization including prescription and non-prescription medication as well as medication or treatment with long half-life or long-term effect. (e.g. immunoglobulins, blood transfusion and immunosuppressants).
(10) Immunization history (i.e. previous immunizations and any adverse event following immunization (AEFI)), in particular occurrence of antenatal bleeding after a previous immunization.

#### 3.1.3 Details of the immunization

For all cases and/or all study participants, as appropriate, the following information should be recorded:

(11) Date and time of immunization(s).
(12) Description of vaccine(s) (name of vaccine, manufacturer, lot number, dose (e.g. 0.25 mL, 0.5 mL, etc.) and number of dose if part of a series of immunizations against the same disease).
(13) The anatomical sites (including left or right side) of all immunizations (e.g. vaccine A in proximal left lateral thigh, vaccine B in left deltoid).
(14) Route and method of administration (e.g. intramuscular, intradermal, subcutaneous, and needle-free (including type and size), other injection devices).
(15) Needle length and gauge.

#### 3.1.4 The adverse event

(16) For all cases at any level of diagnostic certainty and for reported events with insufficient evidence, the criteria fulfilled to meet the case definition should be recorded.

Specifically document:

(17) Clinical description of signs and symptoms of antenatal bleeding, and if there was medical confirmation of the event (i.e. patient seen by physician).
(18) Date/time of onset,^5^ first observation^6^ and diagnosis,^7^ end of episode^8^ and final outcome.^9^
(19) Concurrent signs, symptoms, and diseases.
(20) Measurement/testing.
  • Values and units of routinely measured parameters (e.g. temperature, blood pressure) – in particular those indicating the severity of the event;
  • Method of measurement (e.g. type of thermometer, oral or other route, duration of measurement, etc.);
  • Results of laboratory examinations, surgical and/or pathological findings and diagnoses if present.
(21) Treatment given for antenatal bleeding, including blood transfusion and timing of delivery.
(22) Outcome^8^ at last observation.
(23) Objective clinical evidence supporting classification of the event as “serious”.^10^
(24) Exposures other than the immunization 24 h before and after immunization (e.g. food, environmental) considered potentially relevant to the reported event.

#### 3.1.5 Miscellaneous/ general

(25) The duration of surveillance for antenatal bleeding should be predefined based on specific gestational age at the time of the bleeding event and
  • Biologic characteristics of the vaccine e.g. live attenuated versus inactivated component vaccines;
  • Biologic characteristics of the vaccine-targeted disease;
  • Biologic characteristics of antenatal bleeding including patterns identified in previous trials (e.g. early-phase trials); and
  • Biologic characteristics of the vaccinee (e.g. nutrition, underlying disease like immunodepressing illness).
(26) The duration of follow-up reported during the surveillance period should be predefined likewise. It should aim to continue to resolution of the event.
(27) Methods of data collection should be consistent within and between study groups, if applicable.
(28) Follow-up of cases should attempt to verify and complete the information collected as outlined in data collection guidelines 1–24.
(29) Investigators of patients with antenatal bleeding should provide guidance to reporters to optimize the quality and completeness of information provided.
(30) Reports of antenatal bleeding should be collected throughout the study period regardless of the time elapsed between immunization and the adverse event. If this is not feasible due to the study design, the study periods during which safety data are being collected should be clearly defined.

### 3.2 Data analysis

The following guidelines represent a desirable standard for analysis of data on antenatal bleeding to allow for comparability of data, and are recommended as an addition to data analyzed for the specific study question and setting.

(31) Reported events should be classified in one of the following five categories including the two levels of diagnostic certainty. Events that meet the case definition should be classified according to the levels of diagnostic certainty as specified in the case definition. Events that do not meet the case definition should be classified in the additional categories for analysis.

**Event classification in 5 categories**^11^

**Event meets case definition**
(1) Level 1: *Criteria as specified in the Antenatal Bleeding case definitions.*
(2) Level 2: *Criteria as specified in the Antenatal Bleeding case definitions.*
**Event does not meet case definition**
***Additional categories for analysis***
(3) Reported antenatal bleeding with insufficient evidence to meet the case definition.^12^
(4) Not a case of antenatal bleeding.^13^

(32) The interval between immunization and reported antenatal bleeding (separated out by etiology) could be defined as the date/time of immunization to the date/time of onset^4^ of the first symptoms and/or signs consistent with the definition. If few cases are reported, the concrete time course could be analyzed for each; for a large number of cases, data can be analyzed in the following increments:

**Subjects with Antenatal Bleeding (specify which etiology) by Interval to Presentation**

**Table:** 

**Interval**	**Number**
<24 h after immunization	
24 h to <7 days after immunization	
7 days to <30 days after immunization	
30 days or greater after immunization	
**TOTAL**

These intervals were arbitrarily chosen, as there is no biologic plausibility between vaccination and antenatal bleeding, as we have previously explained. We caution that all episodes of bleeding that occur temporally after vaccination may not be causally linked. For example, pre-existing conditions in pregnancy (i.e., history of abdominal trauma, abnormal placentation, abnormal pregnancy implantation) may predispose a patient to an event of antenatal bleeding, with the administration of a vaccination temporally along the pathophysiologic process to bleeding without any relation.
In addition, we recommend recording both the gestational age at the time of immunization, and the gestational age at the time of the bleeding event. Please refer to the Brighton Collaboration document on establishing gestational age.
(33) The duration of a possible antenatal bleeding event could be analyzed as the interval between the date/time of onset^3^ of the first symptoms and/or signs consistent with the definition and the end of episode^7^ and/or final outcome.^8^ Whatever start and ending are used, they should be used consistently within and across study groups.

(34) If more than one measurement of a particular criterion is taken and recorded, the value corresponding to the greatest magnitude of the adverse experience could be used as the basis for analysis. Analysis may also include other characteristics like qualitative patterns of criteria defining the event.

(35) The distribution of data (as numerator and denominator data) could be analyzed in predefined increments (e.g. measured values, times), where applicable. Increments specified above should be used. When only a small number of cases is presented, the respective values or time course can be presented individually.

(36) Data on antenatal bleeding obtained from subjects receiving a vaccine should be compared with those obtained from an appropriately selected and documented control group(s) to assess background rates of hypersensitivity in non-exposed populations, and should be analyzed by study arm and dose where possible, e.g. in prospective clinical trials.

### 3.3 Data presentation

These guidelines represent a desirable standard for the presentation and publication of data on antenatal bleeding that occurs in a pregnancy in which immunizations are also administered to allow for comparability of data, and are recommended as an addition to data presented for the specific study question and setting. Additionally, it is recommended to refer to existing general guidelines for the presentation and publication of randomized controlled trials, systematic reviews, and meta-analyzes of observational studies in epidemiology (e.g. statements of Consolidated Standards of Reporting Trials (CONSORT), of Improving the quality of reports of meta-analyzes of randomized controlled trials (QUORUM), and of meta-analysis Of Observational Studies in Epidemiology (MOOSE), respectively).

(37) All reported events of antenatal bleeding should be presented according to the categories listed in guideline 31 and 32.

(38) Data on possible antenatal bleeding events should be presented in accordance with data collection guidelines 1–24 (verify numbers) and data analysis guidelines 31–36 (verify numbers).

(39) Terms to describe antenatal bleeding such as “low-grade”, “mild”, “moderate”, “high”, “severe” or “significant” are highly subjective, prone to wide interpretation, and should be avoided, unless clearly defined.

(40) Data should be presented with numerator and denominator (n/N) (and not only in percentages), if available.
Although immunization safety surveillance systems denominator data are usually not readily available, attempts should be made to identify approximate denominators. The source of the denominator data should be reported and calculations of estimates be described (e.g. manufacturer data like total doses distributed, reporting through Ministry of Health, coverage/population based data, etc.).

(41) The incidence of cases in the study population should be presented and clearly identified as such in the text.

(42) If the distribution of data is skewed, median and range are usually the more appropriate statistical descriptors than a mean. However, the mean and standard deviation should also be provided.

(43) Any publication of data on antenatal bleeding should include a detailed description of the methods used for data collection and analysis as possible. It is essential to specify:
• The study design.
• The method, frequency and duration of monitoring for antenatal bleeding.
• The trial profile, indicating participant flow during a study including drop-outs and withdrawals to indicate the size and nature of the respective groups under investigation.
• The type of surveillance (e.g. passive or active surveillance).
• The characteristics of the surveillance system (e.g. population served, mode of report solicitation).
• The search strategy in surveillance databases.
• Comparison group(s), if used for analysis.
• The instrument of data collection (e.g. - questionnaire, diary card, report form).
• Whether the day of immunization was considered “day one” or “day zero” in the analysis.
• Whether the date of onset^4^ and/or the date of first observation^5^ and/or the date of diagnosis^6^ was used for analysis.
• Use of this case definition for antenatal bleeding, in the abstract or methods section of a publication.^14^

## Disclaimer

The findings, opinions and assertions contained in this consensus document are those of the individual scientific professional members of the working group. They do not necessarily represent the official positions of each participant’s organization.

## Footnotes

3 The case definition should be applied when there is no clear alternative diagnosis for the reported event to account for the combination of symptoms.

4 If the reporting center is different from the vaccinating center, appropriate and timely communication of the adverse event should occur.

5 The date and/or time of onset is defined as the time post immunization, when the first sign or symptom indicative for antenatal bleeding occurred. This may only be possible to determine in retrospect.

6 The date and/or time of first observation of the first sign or symptom indicative for antenatal bleeding can be used if date/time of onset is not known.

7 The date of diagnosis of an episode is the day post immunization when the event met the case definition at any level.

8 The end of an episode is defined as the time the event no longer meets the case definition at the lowest level of the definition.

9 E.g. recovery to pre-immunization health status, spontaneous resolution, therapeutic intervention, persistence of the event, sequelae, death.

10 An AEFI is defined as serious by international standards if it meets one or more of the following criteria: (1) it results in death, (2) is life-threatening, (3) it requires inpatient hospitalization or results in prolongation of existing hospitalization, (4) results in persistent or significant disability/incapacity, (5) is a congenital anomaly/birth defect, (6) is a medically important event or reaction.

11 To determine the appropriate category, the user should first establish, whether a reported event meets the criteria for the lowest applicable level of diagnostic certainty, e.g. Level three. If the lowest applicable level of diagnostic certainty of the definition is met, and there is evidence that the criteria of the next higher level of diagnostic certainty are met, the event should be classified in the next category. This approach should be continued until the highest level of diagnostic certainty for a given event could be determined. Major criteria can be used to satisfy the requirement of minor criteria. If the lowest level of the case definition is not met, it should be ruled out that any of the higher levels of diagnostic certainty are met and the event should be classified in additional categories four or five.

12 If the evidence available for an event is insufficient because information is missing, such an event should be categorized as “Reported antenatal bleeding with insufficient evidence to meet the case definition”.

13 An event does not meet the case definition if investigation reveals a negative finding of a necessary criterion (necessary condition) for diagnosis. Such an event should be rejected and classified as “Not a case of antenatal bleeding”.

14 Use of this document should preferably be referenced by referring to the respective link on the Brighton Collaboration website (http://www.brightoncollaboration.org).
